# Human-Inspired Dexterity-Oriented Perception and Trajectory Optimization for Robotic Surface Inspection

**DOI:** 10.3390/biomimetics11050296

**Published:** 2026-04-24

**Authors:** Menghan Zou, Yuchuang Tong, Tianbo Yang, Zhengtao Zhang

**Affiliations:** 1 Institute of Automation, Chinese Academy of Sciences, Beijing 100190, China; zoumenghan2024@ia.ac.cn (M.Z.);; 2 The School of Artificial Intelligence, University of Chinese Academy of Sciences, Beijing 100049, China; 3 CAS Engineering Laboratory for Intelligent Industrial Vision, Beijing 100190, China; 4 Beijing Zhongke Huiling Robot Technology Co., Ltd., Beijing 100192, China

**Keywords:** biomimetic perception, hierarchical trajectory optimization, robotic image acquisition, industrial surface inspection, viewpoint and path planning, posture enhancement network

## Abstract

Industrial surface inspection is fundamental to advanced manufacturing, yet reliable robotic image acquisition in complex geometries remains challenging due to severe occlusions and the inherent trade-off between resolution and coverage. Inspired by human visual inspection behaviors and perception–action coordination mechanisms, this paper proposes a hierarchical trajectory optimization framework for robotic image acquisition based on measured point clouds. Specifically, a multi-constraint preprocessing model is developed to emulate human-like active perception strategies, enabling occlusion-aware viewpoint generation over complex concave and convex surfaces with adaptive camera orientation. Building upon this, a multi-objective trajectory optimization method is introduced to coordinate global coverage and local motion efficiency, jointly optimizing viewpoint sequencing, path length, and motion smoothness hierarchically. To further enhance flexibility in constrained environments, a Pose Reachability Augmented Generative Adversarial Network (PRAGAN) is proposed to learn feasible and adaptable imaging postures under kinematic constraints. Experimental results on an industrial robotic platform equipped with 2D and 3D vision systems demonstrate 100% coverage of key surface areas, a 47.0% reduction in path length, and a 37.5% decrease in solution time compared with the baseline in the physical experiments, while ensuring collision-free operation. Both simulation and real-world experiments validate that the proposed framework effectively captures human-inspired perception and motion coordination, providing a practical and scalable solution for complex industrial surface inspection.

## 1. Introduction

Industrial surface inspection plays a critical role in aerospace, military, and high-end manufacturing industries, where reliable visual data acquisition is essential for assessing structural integrity and surface quality [1,2,3]. With the increasing scale and geometric complexity of industrial components, ensuring accurate and efficient inspection has become increasingly challenging. Traditional fixed-camera inspection systems are inherently limited by restricted viewpoints and often suffer from severe occlusions caused by complex geometries, leading to incomplete coverage and reduced inspection reliability [4,5]. Moreover, these systems are typically tailored to specific workpieces and lack adaptability to diverse and dynamically changing industrial scenarios [6,7,8].

**Figure 1:**
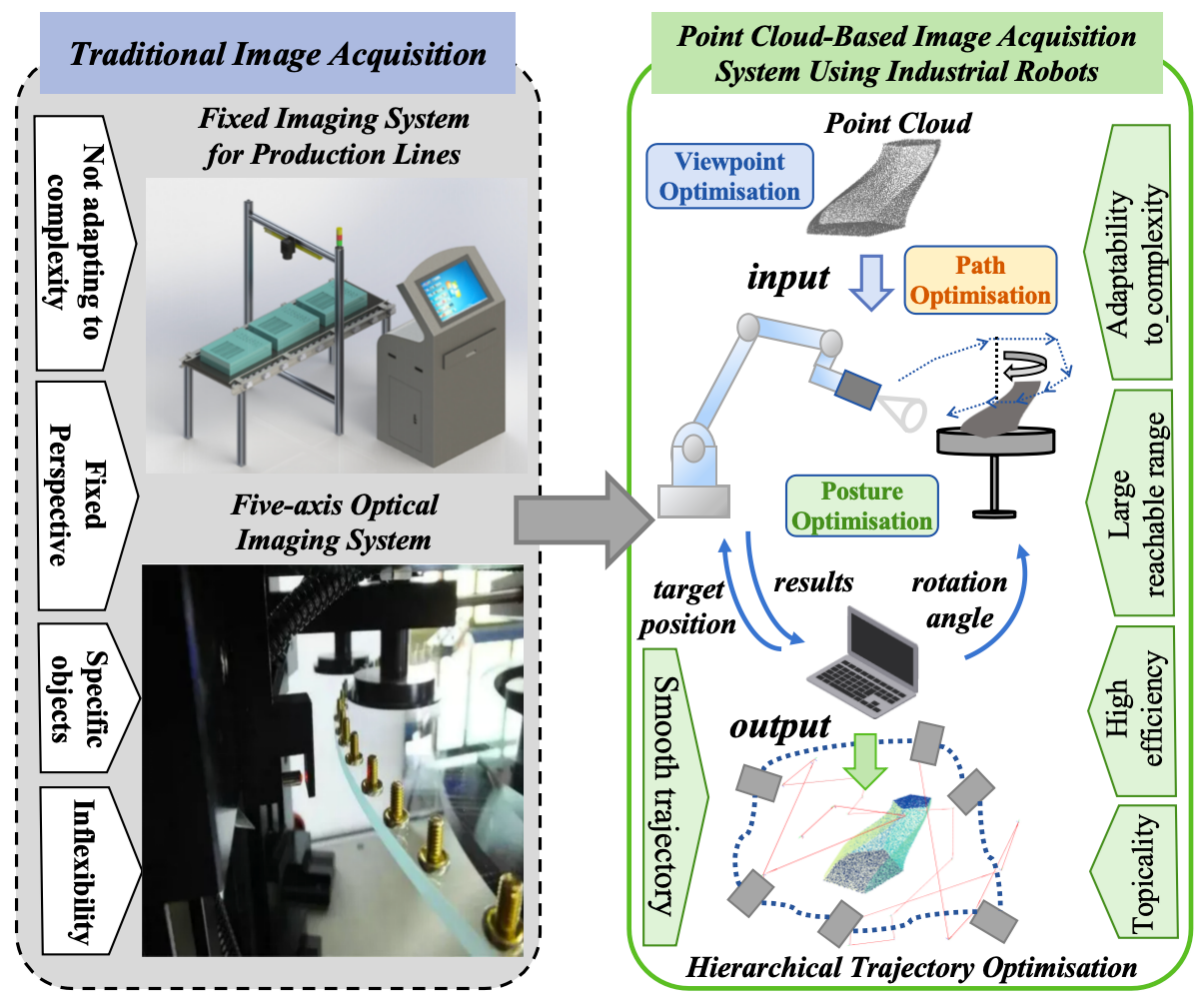
Hierarchical trajectory optimization for industrial robot-based image acquisition systems.

From a biomimetic perspective, human inspectors naturally perform surface inspection through continuous perception–action coordination, actively adjusting their viewpoints and observation angles to handle occlusions and complex structures. This human-inspired active perception mechanism provides important insights for designing more adaptive robotic inspection systems. Recent studies in cognitive science and neuroscience further suggest that biological vision is an active and tightly coupled perception–action process, rather than passive image reception [9,10,11]. In this perspective, the proposed multi-constraint viewpoint preprocessing is analogous to task-directed sensory sampling for acquiring informative and less-occluded observations, the hierarchical trajectory optimization reflects multi-level perception–action coordination during goal-directed behavior, and the PRAGAN-based pose refinement resembles movement/postural adjustment for maintaining perceptual accessibility under kinematic constraints. Industrial robots offer a promising alternative due to their high positioning accuracy and flexible motion capabilities [12,13,14]. By integrating robotic manipulators with vision sensors, robotic inspection systems can emulate such human-like active perception behaviors, dynamically adjusting viewpoints to capture complex surfaces and improve inspection coverage. In particular, point cloud data can be utilized to guide viewpoint planning and trajectory generation, allowing robots to continuously adapt their position and orientation while maintaining appropriate imaging conditions [15,16]. As illustrated in Figure 1, robotic inspection systems enable adaptive image acquisition that improves surface representation accuracy and reduces manual intervention, thereby enhancing inspection efficiency and reliability [17,18].

Despite these advantages, trajectory optimization for robot-based inspection systems remains a challenging problem, especially in complex industrial environments [19,20,21,22,23]. From the perspective of perception–action coupling, inspection planning involves tightly interconnected subproblems, including viewpoint selection, collision-free trajectory generation, and feasible imaging pose determination. These processes are inherently coupled and must be coordinated in a unified manner, similar to how humans integrate visual perception and motion planning during inspection tasks. The problem becomes significantly more complicated when dealing with irregular geometries, cluttered environments, and strict sensing constraints.

Several key challenges arise in practical robotic inspection systems. First, surface irregularities and structural features often introduce severe occlusions, limiting visible regions and complicating viewpoint planning. Selecting appropriate viewpoints that maximize coverage while minimizing occlusion remains a fundamental challenge. Second, inspection planning must balance multiple competing objectives, including coverage completeness, imaging quality, motion efficiency, and collision avoidance. Achieving a globally optimal solution under these constraints is computationally demanding. Third, the feasible imaging pose of the robot is constrained by kinematic reachability and workspace limitations, which further restrict viewpoint selection and trajectory planning [24,25,26]. These challenges highlight the need for a unified framework that can coordinate perception and motion in a human-inspired and adaptive manner.

To address these issues, hierarchical trajectory optimization strategies have recently attracted increasing attention. Instead of treating inspection planning as a monolithic optimization problem, hierarchical approaches decompose the task into multiple stages, such as viewpoint selection, path planning, and pose optimization. This paradigm is consistent with hierarchical decision-making processes observed in biological systems, where global perception and local motion are coordinated across multiple levels. Such a decomposition enables more efficient optimization while improving adaptability in complex environments.

**Figure 2:**
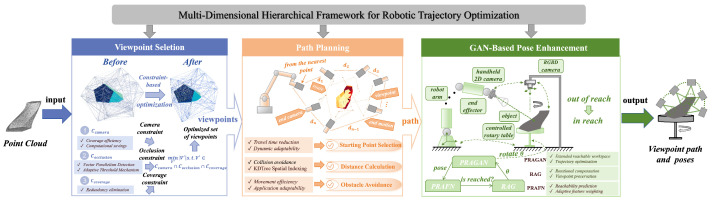
The framework for robot trajectory optimization. Utilizing measured point clouds to facilitate multidimensional optimization of viewpoint selection, path planning, and posture optimization for efficient, precise, and comprehensive image acquisition of complex industrial surfaces.

Motivated by these considerations, this paper proposes a human-inspired hierarchical trajectory optimization framework for robot-based surface inspection. The proposed framework integrates viewpoint selection, trajectory planning, and pose optimization into a unified multi-stage pipeline, enabling efficient and high-quality image acquisition of complex industrial surfaces. Specifically, a multi-constraint viewpoint preprocessing method is first developed to emulate active perception strategies, reducing occlusion and improving the quality of candidate viewpoints. A multi-intelligent trajectory optimization strategy is then introduced to coordinate global coverage and local motion efficiency, generating collision-free and smooth inspection paths. Finally, a Pose Reachability Augmented Generative Adversarial Network (PRAGAN) is proposed to enhance imaging pose feasibility and adaptability under kinematic and environmental constraints. The overall framework is illustrated in Figure 2. Compared with existing inspection planning methods [27,28,29,30,31,32,33,34,35,36], the proposed approach achieves improved inspection coverage, higher planning efficiency, and stronger adaptability to complex industrial environments.

The main contributions of this paper are summarized as follows:

(1) A human-inspired hierarchical trajectory optimization framework that jointly optimizes viewpoint selection, trajectory planning, and imaging pose for efficient robotic surface inspection of complex industrial components.
(2) A multi-constraint viewpoint preprocessing and multi-intelligent trajectory optimization strategy that improves inspection coverage while effectively mitigating occlusions caused by complex geometries, inspired by active perception mechanisms.
(3) A pose reachability augmented generative adversarial network for imaging pose enhancement, improving pose feasibility, adaptability, and imaging quality in constrained industrial environments.

The remainder of this paper is organized as follows. Section 2 reviews related work. Section 3 describes the problem formulation and overall framework. Section 4 presents the viewpoint selection and trajectory optimization methods. Section 5 introduces the PRAGAN-based pose optimization strategy. Section 6 reports experimental validation, and Section 7 concludes the paper.

## 2. Related Work

### 2.1. Surface Inspection and Coverage Planning

Robotic surface inspection has been widely studied in applications such as aerospace inspection, infrastructure monitoring, and industrial quality control. A key problem in these systems is coverage planning, which aims to generate robot trajectories that ensure complete observation of target surfaces. Early approaches mainly relied on geometric decomposition and sensor coverage models [19,20,21,22]. Heuristic optimization methods such as genetic algorithms and ant colony optimization have also been applied to inspection trajectory generation due to their ability to explore large search spaces [37,38,39]. In addition, model predictive control (MPC) and learning-based methods have been explored to improve real-time trajectory generation and adaptability in complex environments [40,41,42,43,44,45].

Despite these advances, most existing studies focus primarily on coverage planning or trajectory optimization independently. The coupling between viewpoint selection, occlusion handling, and robot pose feasibility is rarely considered in a unified framework, which limits the applicability of these approaches in complex industrial inspection tasks.

### 2.2. Occlusion-Aware Viewpoint Planning

Occlusion caused by complex geometries remains a major challenge in robotic visual inspection. Recent studies have increasingly focused on next-best-view (NBV) strategies to improve visibility under occlusion. For instance, gradient-based NBV planning methods have been proposed to efficiently guide viewpoint selection by directly optimizing visibility improvement in local regions [46]. Semantic-aware viewpoint planning approaches further incorporate task-relevant information to prioritize important regions and improve perception accuracy in highly occluded environments [47]. More recent works integrate geometric and semantic constraints to guide viewpoint sampling, such as GS-NBV [48], which improves efficiency by constraining viewpoint search spaces under occlusion. Additionally, projection-based NBV frameworks have been proposed to improve computational efficiency while maintaining coverage performance [49]. Very recent work has further introduced explicitly occlusion-aware NBV models that jointly consider visibility, target completeness, and motion feasibility to improve perception quality in cluttered environments [50].

However, most existing approaches primarily focus on improving visibility or perception quality from a geometric or task-driven perspective. The integration of occlusion-aware viewpoint planning with robot kinematic constraints, trajectory efficiency, and pose feasibility is still limited. As a result, the selected viewpoints may not always be reachable or lead to efficient inspection trajectories in complex industrial scenarios.

### 2.3. Generative Pose Planning

The imaging pose of the robot directly affects inspection quality, sensor visibility, and motion feasibility. Traditional pose optimization approaches often rely on analytical orientation representations such as quaternion-based optimization [51]. Statistical learning methods have also been explored for motion generation. For example, Lim et al. [52] employed principal component analysis (PCA) to synthesize motion patterns from motion capture data. More recently, generative learning models have been introduced to produce candidate robot poses and motions efficiently.

Nevertheless, most existing generative approaches focus on general motion synthesis rather than inspection-oriented pose optimization. They rarely incorporate robot reachability constraints or integrate pose generation with viewpoint selection and trajectory planning, which limits their applicability in robotic surface inspection tasks.

### 2.4. Biological Active Vision and Perception–Action Coordination

Recent biological studies increasingly emphasize that natural vision depends on active sensing and closed-loop interaction with the environment, rather than isolated feedforward perception [9,10,11]. This view provides a biological basis for coupling viewpoint generation, motion sequencing, and pose refinement within a unified hierarchical pipeline. Specifically, viewpoint generation corresponds to active sensory sampling, trajectory sequencing corresponds to organized perception–action coordination across levels, and pose refinement corresponds to adaptive movement adjustment for preserving feasible and informative observation conditions.

## 3. Preliminaries and Overview

### 3.1. Problem Formulation

We formulate the robotic inspection task as a three-stage optimization problem, consisting of viewpoint selection, path planning, and pose refinement.

Given a point cloud of the target surface, the objective is to determine: (1) a minimal set of feasible viewpoints $V^{\prime }$, (2) an optimal visiting sequence P, and (3) a kinematically feasible robot motion defined by $\left(q,\theta_{\mathrm{rot}}\right)$. These stages correspond to visibility feasibility, traversal efficiency, and motion realizability, respectively.

### 3.2. Sequential Optimization Framework

To solve the above problem, we adopt a sequential optimization strategy that decomposes the task into three stages due to its mixed discrete-continuous nature and strong inter-variable dependency. Building upon our previous work [53], we further develop the hierarchical viewpoint planning strategy in this paper as the front-end of a sequential optimization framework. Different from the earlier study, which mainly focused on viewpoint planning itself, the present work further integrates downstream path planning and PRAGAN-based pose optimization under kinematic constraints, thereby forming a complete robotic image-acquisition framework.

Specifically, viewpoint selection first defines a feasible observation space by removing invalid viewpoints under visibility, occlusion, and coverage constraints, which can be formulated as minimizing the number of selected viewpoints $\min_{V^{\prime }}\left|V^{\prime }\right|$. Given the resulting viewpoint set $V^{\prime }$, path planning then determines an optimal visiting sequence by minimizing the total traversal length $\min_{P}L\left(P\right)$ subject to collision-free constraints. Finally, pose refinement ensures that the planned path is physically executable by optimizing robot motion with respect to execution time, collision avoidance, and reachability:

(1) $$\min_{q,\theta_{\mathrm{rot}}}T\left(q,q_{t}\right)+\lambda_{1}f_{\mathrm{col}}-\lambda_{2}\hat{R}\left(q\right)$$

From a unified perspective, the overall objective can be expressed as a weighted combination of viewpoint efficiency, path optimality, and motion feasibility:

(2) $$\min\;\alpha_{1}\left|V^{\prime }\right|+\alpha_{2}L\left(P\right)+\alpha_{3}J_{\mathrm{motion}}$$

In Equation (2), $\alpha_{1}$, $\alpha_{2}$, and $\alpha_{3}$ denote the relative importance of viewpoint compactness, path efficiency, and motion feasibility, respectively. To avoid scale inconsistency among the three terms, $|V^{\prime }|$, L(P), and $J_{\mathrm{motion}}$ are normalized by their corresponding validation-set maxima before weighting. In our implementation, we set $\left(\alpha_{1},\alpha_{2},\alpha_{3}\right)=\left(0.30,0.30,0.40\right)$, where a slightly larger $\alpha_{3}$ is used to emphasize executable and collision-free motion on the physical platform. These values were determined by a coarse validation sweep with a step size of 0.1 under the constraint $\alpha_{1}+\alpha_{2}+\alpha_{3}=1$, and the adopted combination achieved the best overall trade-off between coverage completeness, path length, and motion feasibility.

This sequential decomposition enables tractable optimization at each stage; however, it does not guarantee attainment of the global optimum of Equation (2). The qualitative optimality gap stems from the greedy nature of the pipeline: early-stage viewpoint filtering constrains the candidate set available to downstream path planning, and the final pose-refinement stage optimizes $\left(q,\theta_{\mathrm{rot}}\right)$ over a fixed viewpoint sequence rather than jointly revisiting all previous discrete decisions. Consequently, some globally preferable combinations of viewpoints, visiting order, and poses may be excluded. In return, the decomposition substantially reduces the combinatorial complexity of the mixed discrete–continuous problem, which is critical for real-time inspection. From a feasibility perspective, we do not claim a formal global guarantee for all scenes; instead, feasibility is progressively strengthened across the pipeline. The preprocessing stage removes viewpoints that violate visibility, occlusion, or coverage constraints, the path-planning stage suppresses blocked transitions, and the final pose-refinement stage locally adjusts the motion variables to improve reachability and collision avoidance whenever kinematic adaptation is possible. Therefore, the proposed framework should be viewed as a practical near-optimal engineering strategy that trades possible global optimality loss for the real-time performance and robust executability demonstrated in Section 6.

## 4. Preprocessing and Trajectory Optimization

This section outlines the viewpoint preprocessing model and path planning model employed in the robot trajectory optimization framework.

### 4.1. Multiple-Constraint in Viewpoint Preprocessing

**Figure 3:**
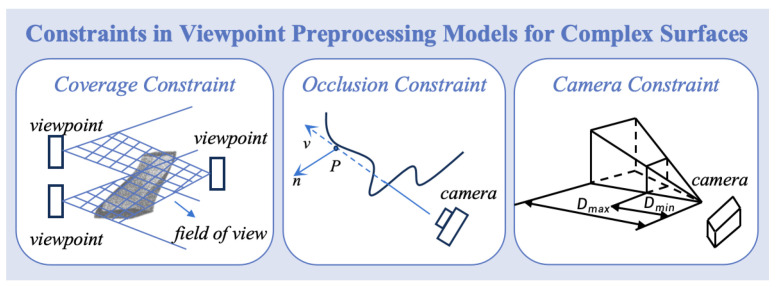
Constraints for the complex surface viewpoint preprocessing model.

The viewpoint preprocessing model, incorporating multiple constraints, optimizes viewpoint selection by addressing occlusions of concave and convex surfaces. This is achieved by selecting optimal viewpoints and adjusting camera angles, as shown in Figure 3.

#### 4.1.1. Camera Constraint

We adopt the pinhole camera model to define the visibility constraint of 3D points, as shown in Algorithm 1. A point $P_{w}$ in the world coordinate system is first transformed into the camera coordinate system:

(3) $$P_{c}=T_{cw}P_{w}$$

where $T_{cw}$ is the extrinsic transformation matrix. Let $P_{c}={\left(x,y,z\right)}^{⊤}$. A point is considered visible if it lies within the camera frustum:

(4) $$\begin{array}{c} \left\{\begin{array}{c} d_{\min}\leq z\leq d_{\max}\\ \left|x\right|\leq z\tan\left(\frac{\theta_{x}}{2}\right)\\ \left|y\right|\leq z\tan\left(\frac{\theta_{y}}{2}\right) \end{array}\right. \end{array}$$

where $\theta_{x}$ and $\theta_{y}$ denote the horizontal and vertical field-of-view (FOV) angles, respectively. The FOV is related to the intrinsic matrix K by:

(5) $$\theta_{x}=2\arctan\left(\frac{W}{2f_{x}}\right),\;\theta_{y}=2\arctan\left(\frac{H}{2f_{y}}\right)$$

**Algorithm 1** Camera Constraint  1:Initialize the unit vector array  2:Generate the transformation matrix  3:Transform the point cloud coordinates to the camera coordinate system  4:Initialize the visible point index list *V*  5:**for** each point $p_{i}$ in the transformed point cloud **do**  6:    Compute the position $\left(x_{i},y_{i},z_{i}\right)$ and depth $d_{i}$ of each point $p_{i}$ in the camera coordinate system  7:    Check if each point $p_{i}$ is within the camera’s field of view  8:    **if** the point $p_{i}$ is within the field of view **then**  9:        Add the index of this point *i* to the visible point index list *V*10:    **end if**11:**end for**12:**return** the visible point index list *V*

#### 4.1.2. Occlusion Constraint

The pseudocode is in Algorithm 2, it is then determined whether occlusion occurs, detailed implementation of the referenced algorithm is as follows:

(6) $$\begin{array}{c} D_{ij}=\frac{\left(v-p_{i}\right)\cdot \left(p_{j}-p_{i}\right)}{∥v-p_{i}∥∥p_{j}-p_{i}∥}>\;\mathrm{limit}\; \end{array}$$

where $p_{1}$ and $p_{2}$ denote any two points satisfying the camera constraints at viewpoint *v*, and limit is the occlusion threshold, set to 0.99 in our experiments; for new targets, 0.99 can be used as a default and slightly adjusted according to point-cloud density and noise level.

This algorithm provides significant advantages in three-dimensional scene analysis. By precisely identifying occluded points, it enhances scene understanding and improves rendering accuracy. The vector-based parallelism detection method is computationally efficient, enabling real-time processing even with dense point clouds. Furthermore, the algorithm’s adaptive threshold mechanism allows for context-specific adjustments, making it suitable for diverse applications from robotics to augmented reality.
**Algorithm 2** Occlusion Constraint  1:Initialize the invisible point index set *I*  2:Construct vectors from the viewpoint to each point  3:**for** each point $p_{j}$ in the point cloud **do**  4:    For each point $p_{j}$, construct the vector from point $p_{j}$ to other points $p_{k}$  5:    Compute the unit vectors of both sets of vectors  6:    Calculate the dot product of the unit vectors  7:    Check if the vectors are approximately parallel  8:    **if** the vectors are parallel **then**  9:        Add to the invisible point index set *I*10:    **end if**11:**end for**12:**return** the invisible point index set *I*

#### 4.1.3. Coverage Constraint

The pseudocode of the proposed method is presented in Algorithm 3, and the key formula involved in the algorithm is given below.

(7) $$\begin{array}{c} S_{\mathrm{final}\;}=S_{v_{1}}\backslash \left\{p\in S_{v_{1}}|\exists v_{2}\in \left(V_{v_{1}}\right)\},p\in V_{v_{2}}\;\mathrm{and}\;p\notin S_{v_{2}}\right\} \end{array}$$

where $v_{1},v_{2}\in V^{\prime }$, and $V_{v_{1}}$ and $V_{v_{2}}$ denote the visible point sets associated with viewpoints $v_{1}$ and $v_{2}$, respectively. Correspondingly, $S_{v_{1}}$ and $S_{v_{2}}$ represent the occlusion sets, and *p* denotes an arbitrary point within the set $S_{v_{1}}$. Starting from the $V_{v_{1}}$ and $S_{v_{1}}$, it is then checked whether *p* is visible from $v_{2}$.

This algorithm provides significant advantages for multi-view camera systems by ensuring optimal coverage of the scene. By systematically evaluating visibility across multiple viewpoints, it eliminates redundant data collection while maximizing scene coverage. The set-theoretic approach efficiently identifies blind spots and optimizes camera placement for comprehensive scene reconstruction. Additionally, the algorithm adaptively balances coverage and computational resources by prioritizing viewpoints that contribute the most unique information.
**Algorithm 3** Coverage Constraint  1:Obtain the camera extrinsic matrix  2:Initialize the visible point list *V* and visible point set *S*  3:**for** each camera position **do**  4:    Filter visible points according to the first constraint  5:    Update the visible point set *S*  6:**end for**  7:Initialize the visible point cloud list *L* and the index list *I*  8:Filter invisible points according to the second constraint  9:**for** each viewpoint $v_{k}$ **do**10:    Obtain the position of $v_{k}$ and the point cloud11:    Call the parallel vector check function12:**end for**13:Determine which points are occluded under all viewpoints14:**return** the final filtered result

### 4.2. Multiple-Constraint in Path Planning

Considering the impact of the actual environment, this section mainly introduces three constraints in path planning to adapt the results to more complex environments.

#### 4.2.1. Starting Point Constraint

Select the viewpoint closest to the robot as the starting point, as follows:

(8) $$\begin{array}{c} \left\{\begin{array}{c} d_{i}=\mathrm{dist}\left(v_{i},P\right)\\ i^{*}=\arg\min_{i}d_{i}\\ k=min\{j|\pi_{j}=i^{*}\}\\ \pi^{\prime }=\left(\pi_{k},\pi_{k+1},...,\pi_{n},\pi_{1},\pi_{2},...,\pi_{k-1}\right) \end{array}\right. \end{array}$$

#### 4.2.2. Distance Constraint

In three-dimensional space, it is crucial to account for both the Euclidean distance and directional disparities. Therefore, taking into account the influence of direction, the distance between two viewpoints is calculated as follows:

(9) $$\begin{array}{c} D=\sqrt{{\left(x_{2}-x_{1}\right)}^{2}+{\left(y_{2}-y_{1}\right)}^{2}+{\left(z_{2}-z_{1}\right)}^{2}}-c\cdot \left(\frac{v_{1}\cdot v_{2}}{∥v_{1}∥∥v_{2}∥}\right) \end{array}$$

where *c* is a directional weighting coefficient that balances Euclidean proximity and viewing-direction consistency. In our implementation, c=0.20m was selected by a validation sweep over {0,0.1,0.2,0.3,0.4}m. Small values make Equation (9) degenerate toward pure Euclidean distance and weaken directional continuity, whereas overly large values over-emphasize orientation agreement and may introduce unnecessary detours. The chosen value provides the best trade-off between path length and viewpoint planning success rate on the validation objects.

#### 4.2.3. Occlusion Constraint

When determining the distance between two viewpoints, it is essential to assess if the line connecting them meets an item. Consequently, we utilize the KDTree algorithm [54] to search along the connecting line segment, sampling points that serve as the centers of spheres with a certain radius, and thereafter determine the presence of any junction locations, as demonstrated below:

(10) $$\begin{array}{cc} \mathrm{blocked} & =\overset{k}{\underset{j=1}{⋁}}\left(∥p_{i}-p_{s}^{\left(j\right)}∥\;\leq r,\;p_{i}\in P\right) \end{array}$$

Here, *r* denotes the radius of the local collision-check sphere. We set r=0.03m according to the point-cloud resolution and the safety margin used in the robot experiment (d=0.05m). A smaller radius tends to miss near-obstacle interference along the line segment, while a larger radius over-prunes feasible viewpoint transitions. This part comprehensively discusses three key constraints in path planning, aimed at enhancing the adaptability of planning outcomes in complex environments.

### 4.3. Viewpoint Preprocessing and Path Optimization

The viewpoint preprocessing stage serves as the foundation of the framework, filtering the initial viewpoint set by enforcing camera (Equation (4)), occlusion (Equation (6)), and coverage constraints (Equation (7)). These constraints ensure physical feasibility, eliminate redundancy and occlusion, and guarantee sufficient surface coverage. The resulting optimized $V^{\prime }$ balances visibility and efficiency, and is passed to the path planning phase for trajectory generation.

Our approach integrates three classical optimization algorithms into a unified multi-intelligence trajectory optimization framework. The greedy algorithm provides an efficient initial solution, the genetic algorithm explores the global solution space to avoid local optima, and simulated annealing refines the path via a temperature-controlled acceptance for gradual convergence. We utilize recent enhancements: improved A* combined with greedy strategies for faster multi-objective optimization [34], multi-population migration in Genetic Algorithm (GA) to maintain diversity [35], and refined initialization with deletion in Simulated Annealing Algorithm (SAA) to reduce computation while ensuring solution quality [36]. This combination achieves high performance with simplicity and efficiency, demonstrating that classical methods can yield state-of-the-art results in robotic path planning. Implementation details follow.

Firstly, based on the multiple constraints (Equations (8)–(10)), a preliminary path is generated using a greedy algorithm [34], as shown below:

(11) $$\left\{\begin{array}{c} \pi_{\mathrm{greedy}}=\left\{p_{0},p_{1},p_{2},\ldots ,p_{n}\right\}\\ i^{*}=\arg\min_{i}[\mathrm{dist}(v_{i},P)-c\cdot \left(\frac{v_{1}\cdot v_{2}}{∥v_{1}∥∥v_{2}∥}\right)+\lambda \cdot {\mathrm{blocked}}_{i}]\\ k=\min\{j|\pi_{j}=i^{*}\}\\ \pi^{\prime }=\left(\pi_{k},\pi_{k+1},\ldots ,\pi_{n},\pi_{1},\pi_{2},\ldots ,\pi_{k-1}\right) \end{array}\right.$$

where $\pi_{\mathrm{greedy}}$ denotes the preliminary path generated by the greedy algorithm.

This path obtained in Equation (11) serves as a starting solution for both GA [35] and SAA [36]. Utilizing this foundation, GA is employed to execute global optimization of the path, consequently identifying a near-optimal path:

(12) $$\left\{\begin{array}{c} F\left(\pi_{i}\right)=\frac{1}{\sum_{j=0}^{n-1}C\left(\pi_{j},\pi_{j+1}\right)}\\ \pi_{\mathrm{GA}}=\mathrm{GA}\left(\pi_{\mathrm{greedy}}\right) \end{array}\right.$$

where $\pi_{\mathrm{GA}}$ is the path after optimization by GA.

Employing SAA [36] to refine the path derived by GA [35] mitigates the risk of local optima and enhances the path’s smoothness and feasibility as follows:

(13) $$\left\{\begin{array}{c} \Delta C=C_{\mathrm{total}}\left(\pi_{\mathrm{new}}\right)-C_{\mathrm{total}}\left(\pi_{\mathrm{GA}}\right)\\ P_{\mathrm{accept}}=\exp\left(-\frac{\Delta C}{T}\right)\\ \pi_{\mathrm{SA}}=\mathrm{SimulatedAnnealing}\left(\pi_{\mathrm{GA}}\right) \end{array}\right.$$

where $\pi_{\mathrm{SA}}$ is the path after optimization by the SAA.

The path optimization phase adopts a multi-intelligence strategy that combines the Greedy algorithm [34], GA [35], and SAA [36]. Let $n=|V^{\prime }|$ denote the number of retained viewpoints after preprocessing. Under fixed GA population size *M*, generation number *G*, and SAA iteration number *I*, the Greedy, GA, and SAA stages scale as $O(n^{2})$, O(GMn), and O(In), respectively. Therefore, the overall path-optimization complexity is $O(n^{2}+GMn+In)$, indicating that the computation grows mainly with the viewpoint count. By reducing redundant viewpoints in advance, the preprocessing stage helps keep the downstream optimization tractable for large industrial components.

**Remark** **1.** *The preprocessing model utilizing three constraint types, alongside multi-intelligence algorithms and intricate environmental constraints for path optimization, facilitates swift and accurate trajectory planning optimization. This guarantees that the trajectory is both the shortest and compliant with several requirements, including perspective coverage and motion smoothness.*

## 5. PRAGAN-Based Pose Enhancement

This section utilizes the viewpoint trajectory output from Section 4 as the foundational motion trajectory for the robotic arm, and develops the PRAGAN network to enhance the pose for the industrial robot imaging system, addressing the challenge of the robotic arm’s restricted reachability. Specific parameters, hyperparameters, and implementation details will be provided in the subsequent experimental sections.

### 5.1. PRAGAN Architecture

**Figure 4:**
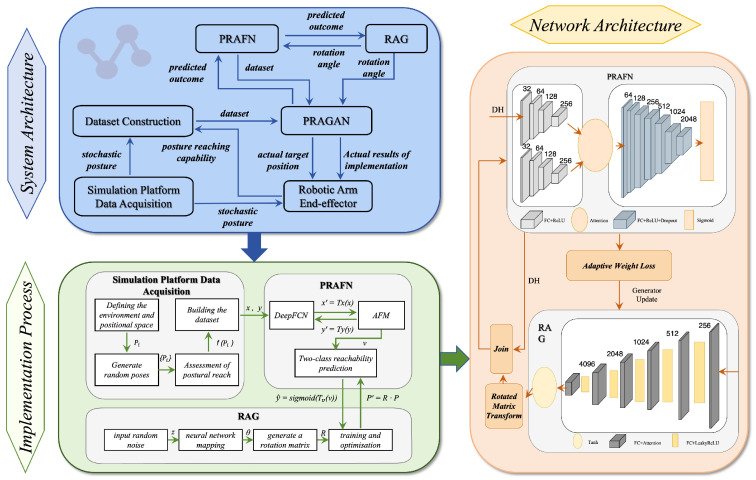
PRAGAN architecture.

The architecture consists of three hierarchical levels that form a comprehensive solution, as illustrated in Figure 4.

**The System Architecture** (top layer—blue) integrates key components: the Simulation Platform Data Acquisition module generates stochastic postures; the Dataset Construction module creates a dataset with reachability information; Pose Reachability Attention Fusion Network (PRAFN) predicts outcomes; RAG generates rotation angles feeding into PRAFN and PRAGAN; and PRAGAN combines these inputs to determine the robotic arm end-effector’s target positions.

**The Implementation Process** (middle layer—green) details the methods: the Simulation Platform defines environment and pose space, generates random postures, and evaluates reachability. PRAFN adopts a dual-path Deep Fully Connected Network (DeepFCN) for pose-feature encoding, followed by an Attention Feature Module (AFM) and a sigmoid-based binary prediction head. Specifically, the two pathways encode the position component ${\vec{p}}_{i}\in R^{3}$ and the orientation component $\mathrm{vec}\left(R_{i}\right)\in R^{9}$ of the pose input $P_{i}=\left({\vec{p}}_{i},R_{i}\right)$, respectively, and the resulting embeddings are fused by AFM. RAG maps random noise to rotation matrices through a neural network optimized via iterative training.

**The Network Architecture** (bottom layer—yellow) presents the neural network structures: PRAFN consists of two fully connected pathways with layer widths {32,64,128,256} and {64,128,256,512,1024,2048}, respectively, followed by AFM-based feature fusion, FC-ReLU layers, and a final sigmoid classifier. RAG uses a fully connected generator with channel dimensions {256,512,1024,2048,4096} and rotated matrix transforms integrated with PRAFN outputs. Attention modules focus computation on critical features, improving pose reachability prediction and rotation angle generation to overcome robotic arm limitations.

This hierarchical approach allows the system to first learn the relationship between poses and reachability, then generate appropriate rotational adjustments, ultimately producing optimized target positions that account for the robotic arm’s kinematic constraints while maintaining the desired viewpoint trajectory. The pseudocode implementation is presented in Algorithm 4, with comprehensive explanations of each algorithmic component elaborated in the following sections.
**Algorithm 4** PRAGAN (PRAGAN)  1:Define large environment domain *D* outside robot workspace  2:Initialize dataset D=∅  3:Pose $P_{i}=\left(\vec{p_{i}},R_{i}\right)$ with position $\vec{p_{i}}$ and rotation $R_{i}$  4:**for** i=1 to *N* **do**  5:    Sample $\vec{p_{i}}∼U\left(a,b\right),\;$θ ∼U(α,β)  6:    Build $R_{i}$ from Euler angles θ, form $P_{i}$  7:    Compute reachability label $f(P_{i})$ via kinematics, store $D\leftarrow (P_{i},f\left(P_{i}\right))$  8:**end for**  9:Initialize discriminator PRAFN with 2 feature pathways + attention fusion10:Train PRAFN by minimizing binary cross-entropy $L_{BCE}$11:Initialize generator RAG (progressive FC layers)12:Train RAG using noise z∼N(0,I) to generate rotation θ, form $P^{\prime }$13:Optimize with GAN losses $L_{D},\;L_{G}$, update generator via $\min L_{G}$14:**return** trained PRAFN and RAG

### 5.2. Simulation Platform Data Acquisition

A location marginally exceeding the robot’s operational range is created on the simulation platform, where random viewpoints are generated. The robot’s accessibility to these viewpoints is documented, generating a collection of viewpoint postures and Boolean indicators of their reachability. The precise steps are as follows.

#### 5.2.1. Definition of Environment and Pose Space

First, a three-dimensional spatial domain *D* is established in the simulation software, with dimensions marginally above the robotic arm’s maximum operational range. This seeks to thoroughly evaluate the operating boundaries of the arm and evaluate its performance under harsh conditions. The spatial coordinates of each point $\left(x,y,z\right)$ and its orientation, denoted by the rotation matrix *R*, collectively produce a pose $P_{i}$.

#### 5.2.2. Random Pose Generation

A script produces a sequence of random poses $\left\{P_{i}\right\}$ inside the spatial domain *D* following a specified distribution to provide uniform coverage of the whole operational space, integrating sufficient variety to evaluate the robotic arm’s adaptation and flexibility. Each pose $P_{i}$ can be mathematically represented by a position vector and a rotation matrix:

(14) $$P_{i}=\left(\vec{p_{i}},R_{i}\right)$$

where $\vec{p_{i}}$ represents the position vector and $R_{i}$ represents the rotation matrix.

The position vector $\vec{p_{i}}=\left(x_{i},y_{i},z_{i}\right)$ defines the coordinates of the end effector’s center point in space. The components $\left(x_{i},y_{i},z_{i}\right)$ are independently sampled from U(a,b):

(15) $$x_{i},y_{i},z_{i}∼U\left(a,b\right)$$

where U(a,b) denotes the uniform distribution over [a,b].

The rotation matrix $R_{i}$ is determined from the Euler angles $\theta_{roll},\theta_{pitch},\theta_{yaw}$ sampled from a uniform distribution:

(16) $$\theta_{roll},\theta_{pitch},\theta_{yaw}∼U\left(\alpha ,\beta \right)$$

where U(α,β) defines the uniform distribution over the interval [α,β] for each Euler angle.

#### 5.2.3. Evaluating Pose Reachability

For each generated posture $P_{i}$, the kinematic model of the robotic arm is used to simulate the transition from its current position to $P_{i}$. The outcomes are denoted by a boolean showing the arm’s ability to attain the target stance. A mapping function f:P→{0,1} is established, with P representing the set of all conceivable positions. For pose $P_{i}$, if accessible, $f(P_{i})=1$; otherwise, $f(P_{i})=0$.

#### 5.2.4. Dataset Construction

The mathematical representation for constructing the dataset is defined as:

(17) $$D=\left\{\left(P_{i},f\left(P_{i}\right)\right)|P_{i}\in P\right\}$$

where P represents the set of all generated poses, and $f(P_{i})$ is the reachability result for pose $P_{i}$. Each $P_{i}$ and its corresponding $f(P_{i})$ are recorded to create a comprehensive dataset. In the implementation used in this paper, the generated pose dataset contains 6.400 randomly generated samples in total, including 3.520 reachable poses and 2.880 unreachable poses, corresponding to a reachable/unreachable ratio of 1.22:1. The dataset was randomly split into 80%/10%/10% for training, validation, and testing, respectively, while preserving the class ratio.

### 5.3. Classifier Training

The assembled dataset is utilized to train the PRAFN employing sophisticated deep learning methodologies to assess the robotic arm’s reachability for designated poses. PRAFN comprises two fundamental components: the Deep Fully Connected Network (DeepFCN) and the Attention Fusion Module (AFM).

Through the incorporation of an attention mechanism, PRAFN dynamically integrates dual input features to augment the recognition of critical information and boost the precision and resilience of pose reachability predictions. The precise steps are as follows:

#### 5.3.1. Data Preprocessing

For each pose $P_{i}=\left({\vec{p}}_{i},R_{i}\right)$, the translational component ${\vec{p}}_{i}={\left[x_{i},y_{i},z_{i}\right]}^{⊤}$ is used as input *x*, while the rotational component is represented by the flattened rotation matrix $\mathrm{vec}(R_{i})$ and used as input *y*. These two inputs are processed by the two pathways of DeepFCN and then delivered to the AFM for feature fusion and subsequent reachability classification.

#### 5.3.2. Attention Mechanism Application

The AFM acquires outputs from both processing pathways of DeepFCN, combines them into a cohesive feature vector via concatenation, computes the relevant attention weights, and utilizes these weights to merge the original inputs. This modifies the contribution ratio of each input, and the weighted outcomes are aggregated to form the fused feature representation. The combination of *x* and *y* is defined as follows:

(18) $$\begin{array}{c} \left\{\begin{array}{cc} c & =[x;y]\\ \alpha & =\mathrm{softmax}(W_{a}\cdot \mathrm{combined}+b_{a})\\ v & =\alpha \cdot x+(1-\alpha )\cdot y \end{array}\right. \end{array}$$

#### 5.3.3. Binary Reachability Prediction

The integrated features are processed via fully connected layers, each succeeded by a ReLU activation function, with the exception of the last layer. The output of the final layer is processed using a Sigmoid function to provide the binary reachability prediction. The training process optimizes the binary cross-entropy loss function defined as:

(19) $$\begin{array}{c} L_{BCE}=-\frac{1}{m}\sum_{i=1}^{m}\left[f\left(P_{i}\right)\log\left(\hat{f}\left(P_{i}\right)\right)+\left(1-f\left(P_{i}\right)\right)\log\left(1-\hat{f}\left(P_{i}\right)\right)\right] \end{array}$$

where *m* is the batch size, $f(P_{i})$ is the true reachability label for pose $P_{i}$, and $\hat{f}\left(P_{i}\right)$ is the predicted probability from PRAFN that pose $P_{i}$ is reachable. Minimizing this loss improves classification accuracy between reachable and unreachable poses.

### 5.4. Generator Training

In PRAGAN, a pre-trained classifier is incorporated as the discriminator PRAFN to evaluate the accessibility of postures suggested by the generator RAG. The RAG generator, structured as a deep neural network, is assigned the objective of learning to produce credible rotation angles θ for the robotic arm from a stochastic noise vector z. The precise steps are as follows:

#### 5.4.1. Random Noise Input

The generator’s input is a random noise vector z sampled from a standard normal distribution $N\left(0,I\right)$, where z∼N(0,I).

#### 5.4.2. Neural Network Mapping

The network processes the noise vector z as input and processes it through a series of fully connected layers $FC_{G}$ and a nonlinear activation function ϕ, resulting in the output of the rotation angle θ, as shown below:

(20) $$\theta =FC_{G}\left(\phi \left(z\right);\Theta_{G}\right)$$

#### 5.4.3. Rotation Matrix Generation

The generated angle θ is transformed into a rotation matrix R(θ), which specifies the transition from the current pose to a new pose. In our experiments, the turntable angle was parameterized within the range θ∈[−π,π], corresponding to $\left[-180^{\circ },180^{\circ }\right]$.

#### 5.4.4. Training and Optimization

Throughout training, the generator and discriminator engage in competition, thereby improving their efficacy. The generator RAG produces a rotation angle for the turntable, which is transformed into a rotation matrix *R* and used to the robotic arm’s pose *P* to obtain the modified pose $P^{\prime }$. This simulates the effect of the turntable on the arm’s position. The modified pose $P^{\prime }$ is next evaluated by the discriminator PRAFN to confirm reachability. The output of the discriminator determines the loss function L, measuring the disparity between $P^{\prime }$ and the actual attainable pose (Equation (21)). Iteratively optimizing this loss enhances RAG, refining the generated rotation angles and ensuring attainable postures.

(21) $$\begin{array}{c} L_{D}=-E_{P_{\mathrm{data}}}\left[\log D\left(P\right)\right]-E_{z∼P_{z}}\left[\log\left(1-D\left(G\left(z\right)\right)\right)\right] \end{array}$$

where $P_{\mathrm{data}}$ represents the distribution of real poses, *z* is a random variable sampled from a noise distribution $P_{z}$, and G(z) is the output of the generator given *z*. The function D(P) represents the discriminator’s output for pose *P*, indicating whether it is real or fake.

Consequently, the loss function for the generator RAG is:

(22) $$L_{G}=-E_{z∼p_{z}}\left[logD\left(G\left(z\right)\right)\right]$$

Throughout the training phase, gradient descent optimizes the loss functions by adjusting the parameters of PRAFN and RAG, enabling RAG to generate more precise turntable angles and allowing PRAFN to better distinguish between authentic poses and those produced by RAG.

**Remark** **2.** *The proposed PRAGAN produces an optimal trajectory that maximizes the arm’s reachability while improving imaging flexibility and accuracy.*

## 6. Experiments

### 6.1. Experimental Setup

This section verifies the correctness and effectiveness of our proposed framework through experiments on simulation and physical platforms, as well as comparative tests. We utilized Python 3.10 and CoppeliaSim 4.6.0 for simulation. All experimental results reported in this paper were obtained on the same industrial control computer equipped with an NVIDIA RTX 4090 GPU, and all compared methods were evaluated under identical hardware conditions to ensure fair comparison. The parameter choices in this study reflect a balance among theoretical considerations, empirical observations, and practical application needs to optimize performance, efficiency, and feasibility. More specifically, several key parameters were determined by preliminary validation before the formal comparative experiments. The directional weighting constant *c*, collision-check radius *r*, and PRAFN classification threshold were set to balance motion efficiency, obstacle screening, feasible path retention, and prediction reliability. The threshold was fixed at 0.7 to ensure robust pose generation and stable image acquisition.

ABCParts [55] is a benchmark dataset comprising diverse and complexly structured mechanical components, from which 10 models were selected as evaluation samples. All experiments in this paper are based on these selected ABCParts objects, and the corresponding physical test pieces were fabricated by 3D printing. These 10 models were selected to cover representative geometric diversity in ABCParts while keeping the full simulation and physical experiments tractable. The experimental parameters are configured as follows: the camera FOV is set to (0.2,0.3) with a depth of field (DOF) of 0.05. The robot arm is initialized at $q\left(0\right)=0_{6\times 1}$ rad, with symmetric joint limits $q^{+}=-q^{-}={\left[\pi ,\pi ,\pi ,\pi ,\pi ,\pi \right]}^{T}$ rad and a minimum collision avoidance distance threshold of d=0.05 m.

The PRAGAN framework adopts a dual-network architecture. The perception network PRAFN employs two parallel fully connected pathways with layer widths {32,64,128,256} and {64,128,256,512,1024,2048}, respectively, followed by AFM-based feature aggregation and a classification threshold of 0.7. The generation network RAG comprises a hierarchical fully connected structure with channel dimensions {256,512,1024,2048,4096}, incorporating FC-Attention and FC-LeakyReLU activations, and outputs the turntable rotation angle θ within the predefined range. Both networks are trained using the Adam optimizer with a learning rate of $2\times 10^{-4}$, weight decay of 0.01, and momentum parameters $\beta_{1}=0.5$, $\beta_{2}=0.999$. PRAFN and RAG were trained for 120 and 80 epochs, respectively, with a batch size of 128. On the NVIDIA RTX 4090 GPU, the offline training of PRAFN and RAG took approximately 4–6 min and 6–10 min, respectively. During training, PRAFN was first pretrained using the pose reachability dataset, after which adversarial optimization was performed. One-sided label smoothing (real label = 0.9) and dropout with a rate of 0.3 were applied to improve training stability. The loss function combines binary cross-entropy for classification with $\ell_{2}$ regularization to mitigate overfitting. The attention modules are configured with a channel reduction ratio of 16 and a temperature parameter of τ=0.07 to improve feature discriminability. For quick reference, the main dataset statistics and PRAGAN training settings are summarized in Table 1. In addition, a brief sensitivity analysis of the key PRAGAN hyperparameters, including the learning rate, batch size, and dropout rate, is provided in Section 6.2.3, and the final setting was selected according to the best balance between convergence stability and orientation optimization performance.

**Figure 5:**
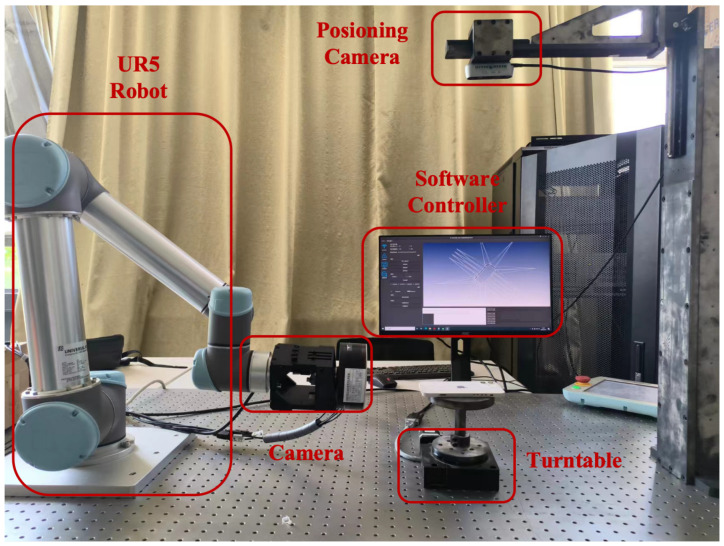
Schematic diagram of the physical platform.

The experimental platform, illustrated in Figure 5, is built upon a UR5e robotic arm as the primary manipulator, equipped with a CCD32-1 industrial camera. The arm’s Denavit–Hartenberg (DH) parameters are specified as: $\alpha ={\left[90,\;0,\;0,\;90,\;-90,\;0\right]}^{T}\;\deg$, $a={\left[0,\;-425,\;-392.25,\;0,\;0,\;0\right]}^{T}\;\mathrm{mm}$, $d={\left[89.459,\;0,\;0,\;109.15,\;94.65,\;82.3\right]}^{T}\;\mathrm{mm}$, and $q={\left[q_{1},\;q_{2},\;q_{3},\;q_{4},\;q_{5},\;q_{6}\right]}^{T}\;\deg$. The setup further incorporates an OPT LT31-1 structured light source, a RealSense D435i depth camera (denoted as the positioning camera in Figure 5) for workspace localization and initial target positioning, and a TBR100 motorized turntable for object repositioning, all coordinated via an industrial control computer. The integration of the turntable introduces an additional redundant degree of freedom into the system. Coupled with the PRAGAN network, this augmentation extends the effective reachability of the manipulator, thereby improving the overall workspace coverage, system scalability, and motion flexibility.

### 6.2. Comparison Experiments

By introducing advanced evaluation metrics [53], this section evaluates the efficacy of viewpoint preprocessing, path planning, and pose optimization, examining their interactions and contributions to framework performance. Furthermore, we evaluate the comprehensive performance of the trajectory optimization framework incorporating these three components against SOTA methods [27,28,29,30].

All stochastic comparative experiments in Section 6.2, Section 6.3 and Section 6.4 were repeated 10 times with different random seeds, and unless otherwise stated, the results of all compared methods were averaged in the same way, i.e., reported as mean ± standard deviation over 10 independent runs. Because the compared optimization procedures contain stochastic components and the sample size is relatively small (n=10), two-sided Wilcoxon signed-rank tests were additionally conducted for the primary paired comparisons. Table 2 summarizes grouped-object descriptive results rather than repeated stochastic trials on the same instance; therefore, no significance test is reported for Table 2.

#### 6.2.1. Viewpoint Preprocessing

In the viewpoint preprocessing phase, we present a constraint-based preprocessing model. To substantiate the merits of this model, we juxtapose it with several widely used preprocessing techniques, including K-Means [31] for clustering, NBV [32] strategy in frontier search algorithms, and the Minimum Spanning Tree (MST) [33] method derived from graph theory. The experiment employs the following evaluation metrics:

- **Crate**: Surface coverage rate (%) achieved by the selected viewpoints.
- ***Vnum***: Number of viewpoints retained after redundancy filtering.
- **Etime**: Execution time (s) of the viewpoint selection algorithm.

**Figure 6:**
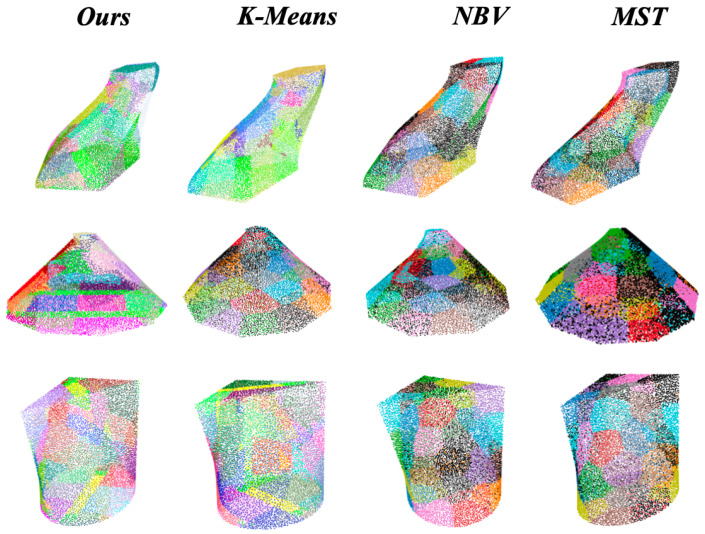
Object coverage with different algorithms [31,32,33]. Distinct colors represent the visible regions from various viewpoints, while black displays the uncovered areas.

In our experiments, we utilized viewpoint preprocessing techniques for various complex objects, categorizing them into three groups according to the principle of analogous initial viewpoint counts, as illustrated in Table 2. The proposed method attained a 100% coverage rate for all three complex objects, providing superior surface coverage relative to methods such as NBV and MST, as depicted in Figure 6. Because this comparison is organized by three object groups with analogous initial viewpoint counts rather than 10 repeated stochastic runs on the same test instance, Table 2 is reported as a descriptive comparison and no hypothesis test is attached to this table.

The NBV method’s coverage rates were slightly lower (94.61%, 94.52%, 94.48%), while the MST method showed variable coverage across objects. In contrast, our method ensures stable and thorough coverage of target objects. Our approach improves efficiency by producing fewer viewpoints (21, 25, 36) compared to K-MEANS (24, 36, 43) and MST (28, 41, 45), reducing redundancy and computational load. This represents nearly a 20% reduction in viewpoints, significantly enhancing execution efficiency. Regarding runtime, our method outperforms K-MEANS and MST, with times of 0.607 s, 0.604 s, and 0.316 s for complex objects one to three. For example, execution on object two is about 76.6% faster than K-MEANS and 97.5% faster than MST. Although NBV runs quickly, it compromises coverage, failing to match our method’s 100% coverage. This highlights our method’s superior balance between real-time performance and comprehensive coverage.

#### 6.2.2. Path Planning

In the viewpoint planning section, a multi-intelligent optimization algorithm is proposed. Its performance is further compared with several representative viewpoint planning approaches, including the Greedy algorithm [34], GA [35], and SAA [36]. The experimental evaluation metrics are defined as follows:

- **PTime**: Planning time (s) for generating a feasible viewpoint sequence.
- ***Pleng***: Total path length, computed as the sum of Euclidean distances between consecutive viewpoints.
- ***NIV***: Number of unreachable viewpoints due to kinematic limitations or collisions.
- **VPSrate**: Viewpoint planning success rate (%), defined as $\frac{1-NIV}{V_{\mathrm{num}}}$.
- **Etime**: Total execution time (s) of the path planning algorithm.

**Figure 7:**
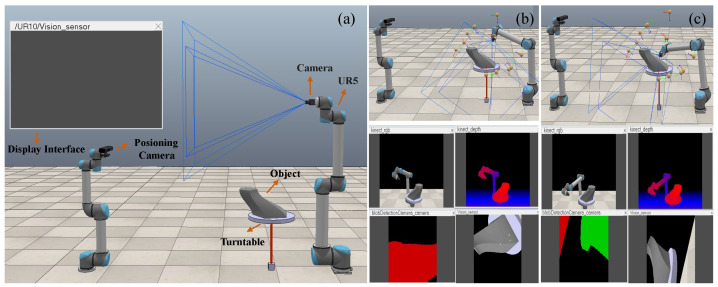
(**a**) Simulation platform. (**b**,**c**) Simulation process.

We developed a simulation platform in CoppeliaSim, integrating our viewpoint planning method to generate motion paths for the robot and to simulate the image acquisition process of the UR5 robot, as depicted in Figure 7. Considering the impact of varying numbers of viewpoints on the complexity of viewpoint planning, we conducted tests on objects with differing numbers of viewpoints, and the results in Table 3 are reported as mean ± standard deviation over 10 independent runs. In our setting, the influence of object size and geometric complexity is reflected by the number of retained viewpoints and traversal length: larger or more complex objects generally require more viewpoints and longer paths, which leads to higher planning time.

**Figure 8:**
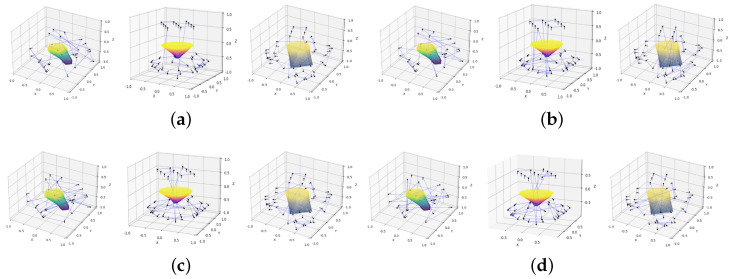
Path planning outcomes for complex objects. (**a**) Ours. (**b**) Greedy [34]. (**c**) GA [35]. (**d**) SAA [36].

The path planning results for different methods applied to complex objects are shown in Figure 8, with complete experimental outcomes from practical tests summarized in Table 3. Our proposed method achieves a runtime comparable to the Greedy algorithm but significantly outperforms both the GA and SAA, reducing planning time to 134.813 s—approximately 14.3% faster than Greedy, 35.6% faster than GA, and substantially faster than the SAA, which requires 478.031 s. The method optimizes the starting point near the robot and incorporates directional differences in distance calculations, resulting in the shortest total path length of 4.721, which is about 35.7% shorter than the Greedy (7.345), GA (8.099), and SAA (10.964) paths. By considering occlusions caused by the object or turntable, our approach minimizes unreachable viewpoints to 0.746, reducing unreachable counts by 54.2%, 42.5%, and 70.8% compared to Greedy, GA, and SAA, respectively. This leads to a high viewpoint planning success rate of 97.51%, exceeding Greedy (94.57%), GA (95.67%), and SAA (91.50%) by over 3%, demonstrating improved coverage and reliability. Furthermore, the method excels in real-time performance, with an execution time of 0.020 s, outperforming Greedy (0.0312 s), GA (0.913 s), and SAA (1.296 s), reflecting over 98% reduction in execution time relative to GA and SAA. Overall, these results confirm that the proposed optimization strategy effectively shortens path length, reduces unreachable viewpoints, improves success rates, and supports efficient real-time execution, making it highly suitable for complex and large-scale robotic path planning tasks. For the primary comparison against the Greedy baseline, the improvement in VPSrate was statistically significant (two-sided Wilcoxon signed-rank test, p=0.0039), and the reduction in Pleng was also statistically significant (p=0.0022).

**Figure 9:**
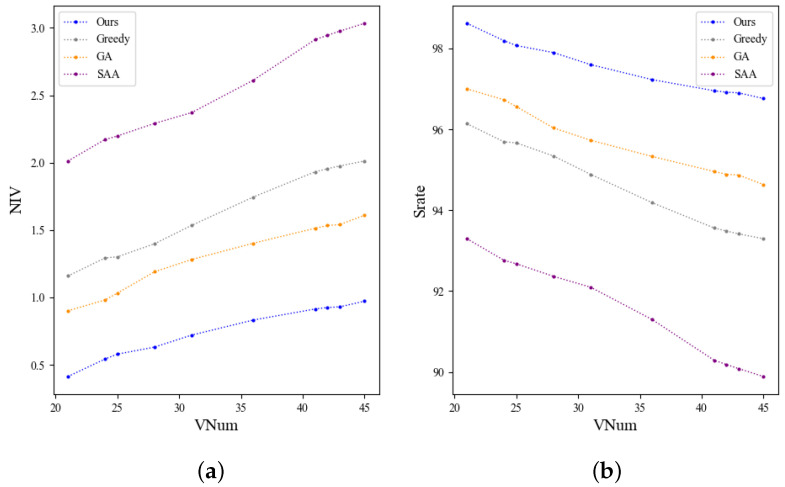
Comparison of viewpoint planning results for objects with varying complexities [34,35,36]. (**a**) NIV. (**b**) Srate.

Figure 9 demonstrates the variations in NIV and Srate among the four methods as the number of viewpoints changes. It can be observed that our algorithm consistently has the lowest number of unreachable viewpoints and the highest success rate in viewpoint planning compared to the other three algorithms. Both metrics gradually stabilize as complexity increases, demonstrating that our algorithm can adapt to various complexity levels and maintains its advantages with highly complex objects.

#### 6.2.3. End-Effector Orientation Optimization

In this section, we verify the performance of the proposed PRAGAN network for end-effector orientation optimization. The core idea is to leverage a physically regularized adversarial network to predict the optimal turntable rotation angle $\theta_{\mathrm{rot}}$ at each viewpoint, replacing the heuristic or exhaustive search strategies adopted by conventional GAN- and VAE-based methods. Specifically, PRAGAN takes as input the current robot joint configuration q and the target surface normal at the planned viewpoint, and outputs a continuous rotation angle that jointly maximizes imaging quality and satisfies kinematic reachability constraints. Compared to standard GANs, PRAGAN incorporates a physics-aware discriminator that penalizes infeasible joint configurations and collision-prone poses during adversarial training, enabling constraint-aware angle generation rather than purely data-driven sampling. Compared to VAE-based approaches, PRAGAN avoids the posterior collapse problem and produces sharper, more discriminative angle distributions, which is critical for precise orientation control in cluttered environments.

We assess how integrating PRAGAN influences the image acquisition system’s performance, using the viewpoint path of a known complex object as a basis for comparison. The evaluation metrics employed in the experiments comprise:

- **ACrate**: Clarity coverage rate (%) of key surface areas.
- ***NIV***: Number of unreachable viewpoints due to kinematic or collision constraints.
- **ColliRate**: Probability of collision (%) during image acquisition.
- **Srate**: Image acquisition success rate (%), defined as achieving ACrate>95% with zero collisions.

**Figure 10:**
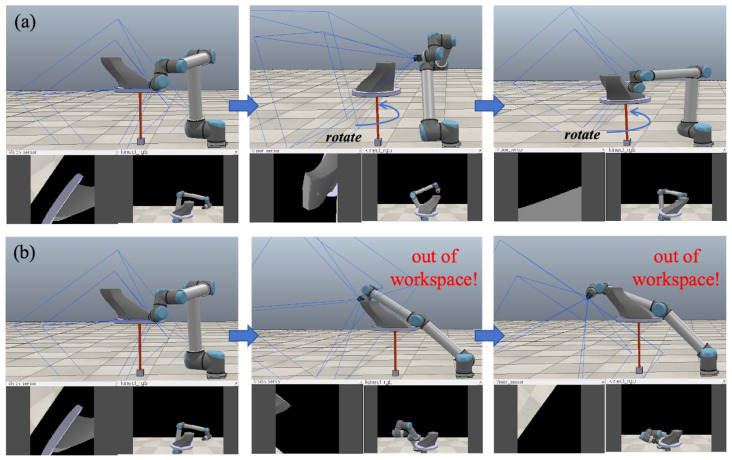
Image acquisition process. (**a**) With PRAGAN. (**b**) Without PRAGAN.

The experimental process is shown in Figure 10. With PRAGAN generating optimal turntable rotation angles, the robotic arm can acquire images from suitable positions. In contrast, without PRAGAN, some viewpoints extend beyond the robotic arm’s workspace, resulting in unreachable positions. As shown in Table 4, integrating PRAGAN significantly improves the image acquisition system’s performance across all metrics. The use of PRAGAN resulted in a clear coverage rate of 98.91% in critical areas, compared to only 90.23% for methods without PRAGAN. This significant enhancement demonstrates that PRAGAN more effectively ensures comprehensive coverage of target areas during viewpoint selection and image capture, substantially improving image quality.

The with-PRAGAN configuration achieved zero unreachable viewpoints, whereas the without-PRAGAN baseline recorded an average of 2.46 unreachable viewpoints per trial, underscoring the efficacy of PRAGAN’s constraint-aware angle prediction in enhancing workspace coverage. Regarding collision safety, PRAGAN reduced the collision rate from 5.36% to 0%, effectively preventing collisions during image acquisition and thereby enhancing the operational reliability of the capturing process. Finally, PRAGAN attained a 100% image acquisition success rate, compared to 89.37% for the baseline, confirming that physics-regularized adversarial training provides a decisive advantage over unconstrained generative models in terms of both quality and safety. A two-sided Wilcoxon signed-rank test further confirmed that the improvement in Srate with PRAGAN was statistically significant (p=0.0034).

We further conducted a brief hyperparameter sensitivity analysis for PRAGAN. With the batch size and dropout rate fixed at 128 and 0.3, respectively, the learning rate of $2\times 10^{-4}$ achieved the best overall performance (ACrate=98.91%, ColliRate=0%, Srate=100%). Reducing the learning rate to $1\times 10^{-4}$ led to slower convergence and slightly lower results (ACrate=98.34%, Srate=99.12%), while increasing it to $5\times 10^{-4}$ introduced mild training oscillation (ACrate=97.85%, ColliRate=0.63%, Srate=98.21%). With the learning rate fixed at $2\times 10^{-4}$ and the dropout rate fixed at 0.3, a batch size of 128 performed better than 64 (ACrate=98.57%, Srate=99.03%) and 256 (ACrate=98.11%, Srate=98.42%), indicating a better trade-off between optimization stability and generalization. With the learning rate fixed at $2\times 10^{-4}$ and the batch size fixed at 128, a dropout rate of 0.3 outperformed 0.1 (ACrate=98.26%, ColliRate=0.41%, Srate=98.76%) and 0.5 (ACrate=97.94%, Srate=98.18%), suggesting that too little dropout caused mild overfitting whereas too much dropout weakened feature learning. These results indicate that the adopted hyperparameter setting provides the most stable overall performance and that PRAGAN remains reasonably robust within a practical parameter range.

Overall, these results validate that PRAGAN’s integration of physical feasibility constraints into adversarial training addresses the fundamental limitations of standard GANs and VAEs in kinematically constrained robotic inspection scenarios, offering a more robust and reliable solution for end-effector orientation optimization.

#### 6.2.4. Hierarchical Trajectory Optimization Framework

In this section, we validate the performance of the proposed hierarchical trajectory optimization framework by comparing it with SOTA methods [27,28,29,30,48,50]. Experiments for different methods were conducted on 10 test samples, and the results in Table 5 are reported as mean ± standard deviation over 10 independent runs. The experimental results are shown in Table 5.

The table shows that our viewpoint preprocessing method reduces Vnum by 18.8%. The decrease in the number of viewpoints indicates that our method effectively removes redundant perspectives, thus improving efficiency and lowering computational complexity. Our method, utilizing the multi-objective balanced path planning component, achieved a ACrate of 100%, surpassing other methods and ensuring comprehensive coverage of the global target area. The Pleng and PTime demonstrate significant superiority compared to alternative methods. The implementation of a posture optimization module has enabled our method to attain a NIV and ColliRate of 0, alongside a Srate of 100%, thereby outperforming all other methods. This demonstrates that our method can ensure high-quality acquisition while guaranteeing the safety of image acquisition. Using the strongest baseline [50] as the primary comparator, the improvement in Srate and the reduction in PTime were both statistically significant (two-sided Wilcoxon signed-rank test, p=0.0137 and p=0.0059, respectively).

This comparative experiment has validated the effectiveness and advancement of the method proposed in this paper.

### 6.3. Ablation Study

This section presents a systematic ablation study to evaluate the contribution of each component within the proposed hierarchical trajectory optimization framework. To ensure statistical reliability, all reported results represent the mean values over 10 independent runs per configuration. Table 6 summarizes the performance of eight module combinations across six metrics: area coverage rate (*ACrate*), path length (*Pleng*), number of invalid viewpoints (*NIV*), collision rate (*ColliRate*), success rate (*Srate*), and planning time (*PTime*).

The baseline configuration, with no modules enabled, achieves an *ACrate* of 89.96% and a *Srate* of 89.36%, but suffers from a high collision rate (5.29%) and long planning time (178.01 s). Incorporating individual modules yields consistent and interpretable improvements: VPM primarily reduces path length and planning time; MIOA improves inspection completeness and suppresses collisions; and PRAGAN eliminates collisions entirely while guaranteeing a 100% success rate. The full integration of all three modules achieves the best performance across all metrics, with 100% coverage, a path length of 1.371, zero collisions, and a planning time of 90.84 s—representing a 49.0% reduction over the baseline. These results confirm the complementary roles of the three modules and validate the overall effectiveness of the proposed framework.

#### 6.3.1. Individual Component Analysis

##### Viewpoint Preprocessing Model (VPM)

The implementation of VPM reduces processing time from 178.01 s to 129.37 s (27.3% reduction) compared to the baseline. Quantitatively, VPM improves area coverage rate from 89.96% to 91.34%, reduces path length from 14.274 to 8.165 (42.8% reduction), and decreases invalid viewpoints from 2.512 to 1.305 (48.0% reduction).

Qualitatively, VPM achieves these improvements through intelligent filtration of viewpoints. By preprocessing the potential viewpoint space and eliminating redundant or sub-optimal perspectives before path planning begins, VPM creates a more focused search space. This preliminary optimization reduces computational overhead in subsequent stages by providing a higher-quality, more manageable set of viewpoints for processing. However, VPM alone cannot completely eliminate invalid viewpoints or collisions, as it does not address robot pose optimization or complex path planning requirements.

##### Multi-Intelligent Optimization Algorithm (MIOA)

When implemented independently, MIOA reduces path length from 14.274 to 4.732 (66.8% reduction) and increases area coverage rate to 93.08%. MIOA also reduces invalid viewpoints by 70.1% and decreases the collision rate from 5.29% to 3.17%.

MIOA achieves these results through sophisticated global and local path planning strategies. By applying a combination of gradient-based and evolutionary algorithms, MIOA effectively balances the competing objectives of coverage maximization, path length minimization, and motion smoothness. However, MIOA’s processing time (136.97 s) is higher than VPM’s because it performs complex multi-objective optimization calculations on the entire viewpoint space. While MIOA significantly improves path efficiency, it cannot completely eliminate reachability issues or collisions without pose optimization.

##### Pose Reachability Augmented GAN (PRAGAN)

PRAGAN demonstrates the most substantial individual impact on reliability, completely eliminating invalid viewpoints and collisions, achieving a perfect success rate of 100%, and increasing area coverage to 98.87%. However, its processing time (150.13 s) is the highest among individual components.

From a qualitative standpoint, PRAGAN accomplishes these results by fundamentally transforming the problem space. Rather than merely planning within constraints, PRAGAN actively modifies the robot’s pose to create feasible solutions where none previously existed. This transformative approach explains both its superior performance in reliability metrics and its higher computational cost. PRAGAN employs advanced deep learning techniques to generate optimal robot poses that adapt to geometric constraints in real-time, effectively solving problems that traditional algorithms consider intractable.

#### 6.3.2. Complete Framework Analysis

##### Integrated Framework Performance

The comprehensive evaluation presented in Table 6 demonstrates the clear performance advantages of the complete framework (VPM + MIOA + PRAGAN), which achieves 100% area coverage and success rate, the shortest path length (1.371), and the lowest processing time (90.84 s), corresponding to a 49.0% reduction compared to the baseline, thereby surpassing all individual and partial module configurations.

##### Qualitative System Analysis

Beyond measurable metrics, the evaluation of each component in Table 6 demonstrates that the complete framework achieves several qualitative improvements that account for its superior and unexpectedly fast performance. The sequential preprocessing by VPM significantly reduces the search space, enabling MIOA to operate with exponentially lower complexity. VPM also provides intelligent initialization, offering near-optimal starting points that accelerate MIOA’s convergence. PRAGAN prevents early failures by filtering out unreachable or collision-prone poses, thereby avoiding wasted computation. Furthermore, the hierarchical structure supports progressive refinement, with each module building on the outputs of the previous stage to minimize resource consumption. This tightly integrated design outperforms any standalone module or partial implementation, as confirmed by ablation studies that reveal substantial reductions in processing time.

### 6.4. Implementation and Verification

**Figure 11:**
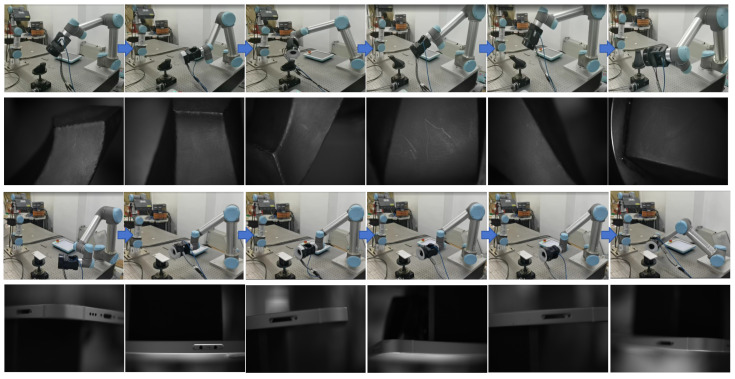
The movement process of the platform and the corresponding captured images.

To further validate the efficacy of our proposed framework in real-world industrial inspections, we conducted a series of appearance inspection experiments targeting both mobile phones and complex objects. Utilizing the inspection platform depicted in Figure 5, the system was designed to traverse a predetermined trajectory, systematically capturing surface images from various viewpoints. The specific inspection process begins with the calibration and configuration of the camera and lighting to ensure optimal image quality. The multi-constraint preprocessing model is employed to identify the best viewpoints, which are then executed through a multi-intelligence trajectory optimization algorithm to generate the inspection path. PRAGAN module enables the robotic arm to capture high-quality images from ideal angles in complex environments. Finally, the captured images can be analyzed for defects, facilitating effective quality control during the manufacturing process. The results, as illustrated in Figure 11, demonstrate that the images obtained are of sufficient clarity to satisfy the stringent requirements necessary for subsequent inspections. This is particularly evident in the case of simple phone frames, where the imaging quality remains consistently high across all captured angles. Moreover, even when inspecting more intricate geometries, the platform successfully maintained high imaging fidelity, especially in critical areas such as corners and edges. This aspect is paramount as it underscores the practical applicability of our method in diverse industrial contexts.

Each physical experiment was also repeated 10 times under the same protocol, and the results in Table 7 are reported as mean ± standard deviation over 10 independent runs. The results presented in Table 7 provide a comprehensive comparison of the performance metrics associated with our proposed inspection framework (referred to as “Ours”) against a baseline method [30] (“Baseline”). Our method achieved a clarity coverage rate of 100%, significantly higher than the 90.37% of the baseline, demonstrating comprehensive coverage of key surface areas. Compared with the baseline, the total path length was reduced from 2537.33 mm to 1344.09 mm, corresponding to a 47.0% reduction. The solution time was reduced from 3049 s to 1907 s, corresponding to a 37.5% decrease. Notably, our approach eliminated unreachable viewpoints, achieving a count of zero, while the baseline had 5.35 unreachable points. The likelihood of collision was also improved to 0%, compared to 2.37% for the baseline, ensuring safe and uninterrupted operations. Furthermore, our method achieved a success rate of image acquisition of 100%, surpassing the baseline’s 89.67%. A two-sided Wilcoxon signed-rank test further showed that the improvement in Srate over the physical baseline was statistically significant (p=0.0031), while the reduction in PTime was also significant (p=0.0044).

**Figure 12:**
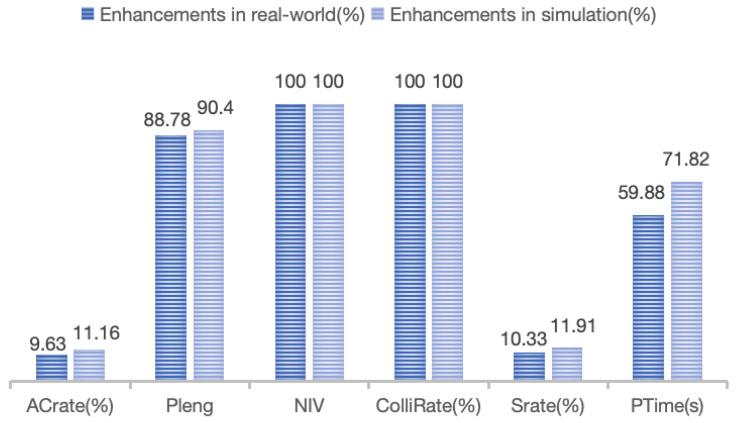
Comparison of enhancement between simulation and real world.

As illustrated in Figure 12, the comparison of enhancement effects between simulation and real-world experiments is demonstrated. Compared with the idealized simulation, the real-world system is additionally affected by camera–robot–turntable calibration errors, point-cloud noise and partial reconstruction uncertainty, illumination variation during image acquisition, and controller latency/mechanical tolerances during execution. These non-ideal factors are not fully modeled in simulation and may lead to slight deviations in viewpoint realization and motion efficiency, thereby causing the performance gap between simulation and physical experiments, although the overall improvement trend remains consistent. Collectively, these results underscore the effectiveness and practicality of our framework in enhancing industrial inspection processes.

The proposed general image acquisition system for complex surfaces demonstrates strong effectiveness in industrial inspection by enabling the capture of detailed images of intricate geometries, essential for defect detection and quality control. Integrating advanced imaging technologies with intelligent trajectory optimization algorithms, the system supports thorough inspections even in challenging environments, with potential applications extending from manufacturing and aerospace to medical devices and pharmaceuticals. The current experimental validation is limited to the UR5e-based inspection platform with a motorized turntable. Nevertheless, the proposed framework is not inherently restricted to this specific hardware configuration. For deployment on a new manipulator or mobile platform, the geometry-driven viewpoint preprocessing and path planning modules can be largely retained after system recalibration, kinematic model substitution, and workspace reconstruction. In contrast, the PRAGAN module depends on platform-specific reachability characteristics and therefore requires corresponding reachability data and dataset adaptation, followed by fine-tuning or retraining. As potential extensions, the framework could be integrated with mobile manipulators or rail-guided systems to enlarge the effective workspace. The viewpoint planning and trajectory optimization modules could also be reformulated in a global coordinate frame without object rotation, which may support the inspection of large and static structures while preserving the core advantages of the proposed method. In this process, human intervention is limited to the initial calibration/configuration and target loading, after which viewpoint generation, path planning, turntable adjustment, and image acquisition are executed automatically; the required learning is performed offline and does not affect the online inspection cycle, with fine-tuning only needed when the manipulator or workspace changes significantly.

## 7. Conclusions

This paper presents a human-inspired hierarchical trajectory optimization framework for robotic image acquisition in complex industrial surface inspection, leveraging biomimetic perception–action coordination mechanisms. By integrating occlusion-aware viewpoint selection, collision-free trajectory planning, and adaptive pose optimization, the framework emulates human-like active perception and motion strategies. Experimental results in the physical experiments, compared with the baseline, demonstrate 100% coverage of key surface areas, a 47.0% reduction in path length, a 37.5% decrease in solution time, fully collision-free operation, and a 100% success rate in high-clarity image acquisition, validating its efficiency, robustness, and reliability. The proposed approach highlights the potential of biomimetic strategies to enhance robotic inspection performance, offering a scalable solution for applications in aerospace, precision manufacturing, and infrastructure maintenance. Future work will focus on integrating learning-based methods to further improve real-time adaptability and generalization in dynamic industrial environments, inspired by biological intelligence.

## Figures and Tables

**Table 1:** Training Configuration.

Item	Setting
Total pose samples	6400
Reachable/unreachable samples	3520/2880
Train/val/test split	80%/10%/10%
Optimizer	Adam
Learning rate	$2\times 10^{-4}$
Weight decay	0.01
$\beta_{1},\beta_{2}$	0.5, 0.999
Batch size	128
PRAFN epochs	120
RAG epochs	80
Classification threshold	0.7
Dropout rate	0.3

**Table 2:** Viewpoint preprocessing comparative experiment.

Objects	Group One			Group Two			Group Three		
Method	Crate (%)	Vnum	Etime (s)	Crate (%)	Vnum	Etime (s)	Crate (%)	Vnum	Etime (s)
Ours	100	21	0.607	100	25	0.604	100	36	0.316
K-MEANS [31]	100	24	1.853	98.30	36	2.580	100	43	2.523
NBV [32]	94.61	25	0.478	94.52	31	0.659	94.48	42	0.239
MST [33]	97.20	28	22.703	93.5	41	24.075	95.39	45	24.471

**Table 3:** Path planning comparative experiment.

Method	Ours	Greedy [34]	GA [35]	SAA [36]
PTime (s)	134.813 ± 4.286	157.314 ± 5.107	209.196 ± 6.942	478.031 ± 13.685
Pleng	4.721 ± 0.193	7.345 ± 0.254	8.099 ± 0.287	10.964 ± 0.413
NIV	0.746 ± 0.118	1.631 ± 0.164	1.298 ± 0.152	2.552 ± 0.241
VPSrate (%)	97.51 ± 0.84	94.57 ± 1.06	95.67 ± 0.91	91.50 ± 1.34
ETime (s)	0.020 ± 0.003	0.0312 ± 0.0041	0.913 ± 0.052	1.296 ± 0.087

**Table 4:** Orientation optimization comparative experiment.

Method	*ACrate* (%)	*NIV*	*ColliRate* (%)	*Srate* (%)
with-PRAGAN	98.91 ± 0.38	0.00 ± 0.00	0.00 ± 0.00	100.00 ± 0.00
without-PRAGAN	90.23 ± 0.74	2.46 ± 0.31	5.36 ± 0.82	89.37 ± 0.142

**Table 5:** Hierarchical trajectory optimization framework comparative experiment.

Method	*Vnum*	*ACrate* (%)	*Pleng*	*NIV*	*ColliRate* (%)	*Srate* (%)	*PTime* (s)
Ours	30.46 ± 0.83	100.00 ± 0.00	1.408 ± 0.071	0.00 ± 0.00	0.00 ± 0.00	100.00 ± 0.00	92.18 ± 3.21
[27]	37.50 ± 1.12	95.31 ± 0.62	3.101 ± 0.116	0.319 ± 0.052	0.00 ± 0.00	98.36 ± 0.057	127.33 ± 4.83
[28]	37.50 ± 1.08	94.03 ± 0.75	2.537 ± 0.103	0.207 ± 0.041	4.97 ± 0.61	97.62 ± 0.094	109.78 ± 4.27
[29]	37.50 ± 1.15	94.37 ± 0.68	3.509 ± 0.129	0.295 ± 0.048	5.29 ± 0.72	97.51 ± 0.046	138.01 ± 5.14
[30]	37.50 ± 0.94	97.46 ± 0.37	2.037 ± 0.082	0.075 ± 0.019	0.00 ± 0.00	99.13 ± 0.013	113.59 ± 3.76
[48]	35.18 ± 0.97	97.88 ± 0.35	2.118 ± 0.087	0.069 ± 0.018	0.00 ± 0.00	99.24 ± 0.015	116.84 ± 3.95
[50]	34.72 ± 0.93	98.03 ± 0.33	2.086 ± 0.084	0.058 ± 0.016	0.00 ± 0.00	99.31 ± 0.013	115.27 ± 3.68

**Table 6:** Ablation study on proposed framework. Gray-shaded cells with check marks indicate enabled modules.

VPM	MIOA	PRAGAN	*ACrate* (%)	*Pleng*	*NIV*	*ColliRate* (%)	*Srate* (%)	*PTime* (s)
			89.96 ± 0.81	14.274 ± 0.436	2.512 ± 0.214	5.29 ± 0.63	89.36 ± 0.147	178.01 ± 6.85
√			91.34 ± 0.74	8.165 ± 0.391	1.305 ± 0.188	5.03 ± 0.58	90.03 ± 0.094	129.37 ± 5.21
	√		93.08 ± 0.69	4.732 ± 0.247	0.751 ± 0.101	3.17 ± 0.41	91.87 ± 0.162	136.97 ± 5.73
		√	98.87 ± 0.33	6.134 ± 0.284	0.00 ± 0.00	0.00 ± 0.00	100.00 ± 0.00	150.13 ± 6.12
√	√		94.31 ± 0.55	3.218 ± 0.186	0.601 ± 0.087	2.78 ± 0.36	92.13 ± 0.018	110.56 ± 4.96
√		√	98.95 ± 0.27	5.960 ± 0.213	0.00 ± 0.00	0.00 ± 0.00	100.00 ± 0.00	119.73 ± 4.43
	√	√	100.00 ± 0.00	1.662 ± 0.074	0.00 ± 0.00	0.00 ± 0.00	100.00 ± 0.00	130.71 ± 5.08
√	√	√	100.00 ± 0.00	1.371 ± 0.061	0.00 ± 0.00	0.00 ± 0.00	100.00 ± 0.00	90.84 ± 3.87

**Table 7:** Results of physical experiments.

	*ACrate* (%)	*Pleng* (mm)	*NIV*	*ColliRate* (%)	*Srate* (%)	*PTime* (s)
Ours	100.00 ± 0.00	1344.09 ± 36.52	0.00 ± 0.00	0.00 ± 0.00	100.00 ± 0.00	1907 ± 84.31
without Ours [30]	90.37 ± 1.14	2537.33 ± 71.68	5.35 ± 0.74	2.37 ± 0.53	89.67 ± 0.192	3049 ± 103.57

## Data Availability

Dataset available on request from the authors.

## Abbreviations

The following abbreviations are used in this manuscript:

PRAGAN	Pose Reachability Augmented Generative Adversarial Network
PRAFN	Pose Reachability Attention Fusion Network
VPM	Viewpoint Preprocessing Model
MIOA	Multi-intelligence Optimization Algorithm
NBV	Next Best View
GA	Genetic Algorithm
SAA	Simulated Annealing Algorithm
FOV	Field of View
